# What Is the Impact of Antimicrobial Photodynamic Therapy on Oral Candidiasis? An In Vitro Study

**DOI:** 10.3390/gels10020110

**Published:** 2024-01-29

**Authors:** Emira D’Amico, Silvia Di Lodovico, Tania Vanessa Pierfelice, Domenico Tripodi, Adriano Piattelli, Giovanna Iezzi, Morena Petrini, Simonetta D’Ercole

**Affiliations:** 1 Department of Medical, Oral and Biotechnological Sciences, University G. d’Annunzio of Chieti-Pescara, 66100 Chieti, Italy; emira.damico@unich.it (E.D.); tania.pierfelice@unich.it (T.V.P.); gio.iezzi@unich.it (G.I.); simonetta.dercole@unich.it (S.D.); 2 Department of Pharmacy, University G. d’Annunzio of Chieti-Pescara, 66100 Chieti, Italy; silvia.dilodovico@unich.it; 3 School of Dentistry, Saint Camillus International University of Health and Medical Sciences, 00131 Rome, Italy

**Keywords:** photodynamic therapy, aminolaevulinic acid, *Candida albicans*, gingival fibroblasts

## Abstract

This study aimed to evaluate the ability of photodynamic therapy, based on the use of a gel containing 5% delta aminolaevulinic acid (ALAD) for 45′ followed by irradiation with 630 nm LED (PDT) for 7′, to eradicate *Candida albicans* strains without damaging the gingiva. *C. albicans* oral strains and gingival fibroblasts (hGFs) were used to achieve these goals. The potential antifungal effects on a clinical resistant *C. albicans S5* strain were evaluated in terms of biofilm biomass, colony forming units (CFU/mL) count, cell viability by live/dead analysis, and fluidity membrane changes. Concerning the hGFs, viability assays, morphological analysis (optical, scanning electronic (SEM), and confocal laser scanning (CLSM) microscopes), and assays for reactive oxygen species (ROS) and collagen production were performed. ALAD-mediated aPDT (ALAD-aPDT) treatment showed significant anti-biofilm activity against *C. albicans S5*, as confirmed by a reduction in both the biofilm biomass and CFUs/mL. The cell viability was strongly affected by the treatment, while on the contrary, the fluidity of the membrane remained unchanged. The results for the hGFs showed an absence of cytotoxicity and no morphological differences in cells subjected to ALAD-aPDT expected for CLSM results that exhibited an increase in the thickening of actin filaments. ROS production was augmented only at 0 h and 3 h, while the collagen appeared enhanced 7 days after the treatment.

## 1. Introduction

The increasingly widespread problem of antibiotic and antifungal resistance demands the development of new alternative treatments [1,2]. Fungi are commensal organisms in the oral microbiome, and the over-presence of such microorganisms or the expression of virulence factors could cause pathological conditions. In addition to the genera *Cryptococcus* and *Aspergillus*, *Candida albicans* is a commensal yeast of the oral cavity that is also able to induce invasive forms of candidiasis in other districts of the body. *C. albicans* is common and underdiagnosed among older people who wear dentures. A good oral care regimen may represent a valid prevention method [3]. Furthermore, *C. albicans* can also indicate systemic disease, such as diabetes mellitus and Cushing syndrome [3]. *C. albicans* also represents a risk factor for oral squamous cell carcinoma (OSCC) due to its potential to induce the production of carcinogenic compounds, such as endogenous nitrosamines. Specifically, *C. albicans* can convert nitrite and/or nitrate into nitrosamines to produce acetaldehyde, which has a carcinogenic role in the oral cavity. *C. albicans* showed the highest nitrosation potential compared to other strains such as *C. tropicalis*, *C. parapsilosis*, and *C. torulopsis* [4].

The pathogenic form of *C. albicans*, which is responsible for oral candidiasis, is produced by the transformation from the yeast state to the pseudohyphoid state because of chemical–physical or microbiological variations in the environment or immunosuppression state [5,6]. The traditional treatment for candidiasis includes the use of antifungal drugs such as amphotericin B, fluconazole, and nystatin. Sometimes, these treatments are not efficacious for infections associated with yeast biofilm, which result in treatment difficulties [7]. Therefore, the development of an alternative treatment is needed. Photodynamic therapy has already shown promising effects on health [8]. The most common photosensitizers include delta aminolaevulinic acid, toluidine blue, and methylene blue [9]. In detail, photodynamic therapy (ALAD-mediated aPDT, ALAD-aPDT), based on the use of 5% 5-aminolaevulinic acid gel (ALAD) and irradiation of 630 nm LED (PDT), has already demonstrated antibacterial effects against Gram-positive (*Enterococcus faecalis*, *Staphylococcus aureus*) and Gram-negative (*Porphromonas gingivalis*, *Veilonella parvula*, *Escherichia coli*) bacteria [10,11]. In addition, Petrini M. et al. demonstrated the ability of ALAD-aPDT to counteract *Streptococcus oralis* biofilm deposited on titanium surfaces with different tomography [12]. The protocol’s effectiveness has been observed in endodontics, showing potent antibacterial activity against *E. faecalis* in infected root canals [13]. The mechanism of action of ALAD-aPDT against bacteria is based on the production of high levels of reactive oxygen species (ROS), which induce irreversible damage to the bacteria [14]. In detail, the application of 5-aminolaevulinic acid (5-ALA), as the precursor of the photosensitizer protoporphyrin IX (PpIX) in the heme pathway, leads to the accumulation of PpIX inside bacteria because of the lack of the specific enzyme ferrochelatase, with consequent production of ROS and cytotoxic effects [14]. In addition, Greco G. et al. observed the inhibition of *C. albicans’* growth in vitro on both biofilm and inoculum via microscopic analysis [15]. However, the absence of toxicity in the surrounding tissues is required. Several studies have demonstrated the ability of ALAD-aPDT to induce proliferative effects in gingival fibroblasts and oral osteoblasts [10,16]. In addition, the ALAD-aPDT enhanced alkaline phosphate (ALP) activity and mineralized deposition of human oral osteoblasts, highlighting a promising potential for bone tissue regeneration [16]. The mechanism of action on mammalian cells is still unknown. Pierfelice TV et al. showed that ROS levels increase quickly after the treatment, but returned to normal after 24 h [16]. This indicates that the mechanism is correlated with ROS generation. Therefore, the first objective of this research was to evaluate the potential antifungal effects on a clinically resistant *C. albicans* strain in terms of biofilm and fluidity membrane changes. The second aim involved evaluating the impact on human gingival fibroblasts (hGFs), investigating the viability, morphological changes, ROS, and collagen production.

## 2. Results and Discussion

### 2.1. Biofilm Biomass Assay

**Figure 1:**
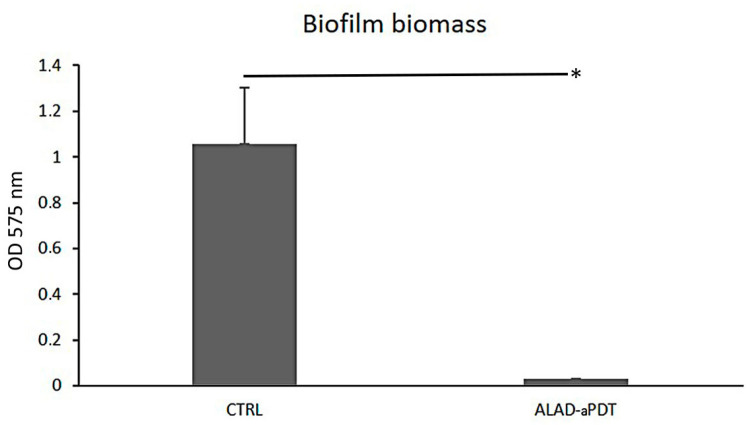
Biofilm biomass (OD_570_) of clinical resistant *Candida albicans S5* strain biofilm after exposure to ALAD-aPDT treatment; CTRL = control, unexposed *C. albicans S5*; * *p*-value < 0.05.

ALAD-aPDT treatment showed a significant anti-biofilm activity against *C. albicans S5*. The biofilm biomass (Figure 1) significantly decreased, *p* < 0.050, for cells exposed to the clinically resistant *C. albicans S5* strain, with biomass values (OD_570_) of 1.06 ± 0.25 and 0.03 ± 0.00 in exposed and unexposed cells, respectively. ALAD-aPDT treatment caused a reduction in fungal biofilm biomass by 97.10% compared to unexposed cells. This outcome was in line with a recent study in which Greco et al. observed an 80% inhibition of growth for biofilm treated with PDT mediated by 5-ALA [15]. Previous studies explored photodynamic inactivation mediated by other photosensitizers combined with red light against fungal biomass. Methylene blue (MB) or 1,9-dimethyl MB (DMMB) combined with a red LED resulted in the effective reduction of *C. auris* by 91% [17]. De Carvalho Leonel et al. applied PDT mediated by MB against oral fungi, and they observed reductions of 37% to 74% for *C. albicans* in both planktonic and biofilm phases in a concentration-dependent manner [18]. The formation of biofilm represents a major form of virulence for *C. albicans*, which often displays tolerance to conventional antifungal treatments. The ability of the ALAD-aPDT photodynamic protocol to counteract and reduce biofilms in vitro, with percentages that are higher than for photodynamic protocols mediated by other photosensitizers previously described, could suggest that the absorption of 5-ALA is higher than methylene blue. The results of this study could form the basis for further investigations concerning the uptake of 5-ALA by yeast. Indeed, 5-ALA is not the photosensitizer itself; the accumulation of PpIX inside the cells could be further investigated.

### 2.2. Colony Forming Units Count

**Figure 2:**
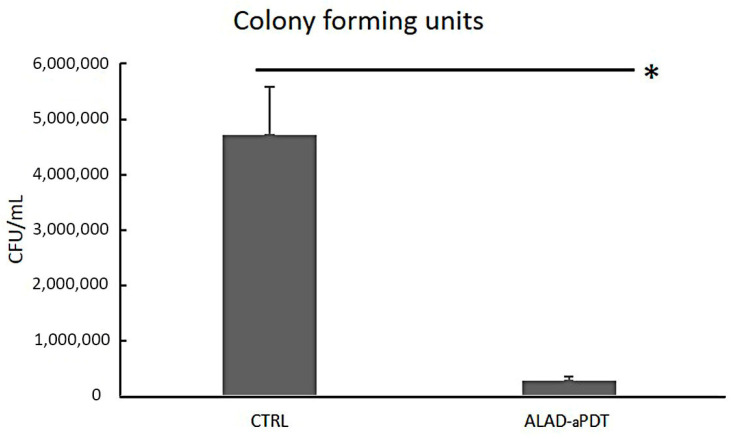
Colony forming units of clinical *Candida albicans S5* strain biofilm after exposure to ALAD-aPDT treatment; CTRL = control, unexposed *C. albicans S5*. * *p*-value < 0.05.

The microbiological evaluation displayed a significant effect of ALAD–aPDT on *C. albicans* biofilm. The treatment demonstrated a significant decrease in fungal load with statistically significant differences compared to untreated cells, *p* < 0.05. In particular, the adherent *C. albicans S5* CFU/mL decreased from 4.72 × 10^6^ ± 8.72 × 10^5^ to 2.73 × 10^5^ ± 8.04 × 10^4^, with a percentage reduction of 94.21 CFU/mL with respect to the control (Figure 2). Other studies have reported the effect of PDT protocols for eliminating oral fungi. However, comparisons are quite difficult to make due to the different photosensitizers, molar concentrations, light sources, wavelengths, and doses. Souza et al. showed that the in presence of methylene blue (MB) (100 g/mL) as the photosensitizer and red laser, the CFU/mL counts decreased by 88.6% for *Candida albicans* [19]. Pupo et al. showed reductions of 62.18% and 73.58% in *C. albicans* CFU/mL counts by applying MB and toluidine blue (TB) and laser irradiation, respectively [20]. The higher reduction in CFU counts could be due to the greater solubility of the 5-ALA gel that permits it to cross the cell membrane more easily than MB and TB. There is little evidence of using 5-ALA in PDT to treat *C. albicans*. Despite the reported decrease in *C. albicans* growth, this happened after extended incubation periods, irradiation exposure, or high ALA concentrations. Monfrecola et al. completely inhibited the in vitro planktonic growth of *C. albicans*; however, this was carried out by incubating the 5-ALA at a concentration of 600 mg/mL (60% 5-ALA) for 3 h, which exceeded the in vivo tolerance limit of 20% ALA-aPDT reported in the literature [21]. Calzavara et al. performed an in vivo study by topically administrating a 20% 5-ALA cream on skin lesions of nine patients with interdigital mycoses of the foot. The medication was applied 4 h before irradiation [22]. Oriel and Nitzan observed a substantial decrease in *C. albicans* planktonic growth when treated with 5-ALA at a dosage of 100 mg/mL (10% 5-ALA) [23]. The results of this study are in accordance with those of Greco et al. who used the same gel with aminolevulinic acid used in this study. They showed a growth inhibition of CFUs in both *Candida* biofilm and inoculum by ∼80% and ∼95%, respectively, after 1 h of incubation and 7 min of red-light irradiation. [15] The same authors showed that the antifungal activity was also due to the 3.5 pH level of the ALAD gel.

Applying a photodynamic protocol mediated by a gel containing 5-ALA in a concentration of 5% (5% 5-ALA) incubated for 45 min and triggered with 630 nm light applied for 7 min emphasized the significance of our findings.

### 2.3. Live/Dead

The images obtained using fluorescent microscopy demonstrated that ALAD–aPDT exerted a significant antifungal effect with respect to the unexposed cells.

**Figure 3:**
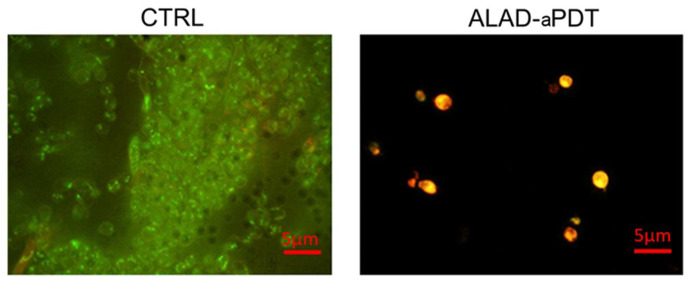
Live/dead staining of *Candida albicans S5* exposed to ALAD-aPDT. Fluorescent representative images show viable (green) and dead (red) cells after treatment with ALAD-aPDT with respect to the unexposed ones.

The live/dead assay showed that 100% of fungal cells exposed to ALAD–aPDT were red (dead) in comparison to the 90% of unexposed cells that were green (live) (Figure 3). This result confirmed the previous results concerning the CFU counts.

Since the *C. albicans* clinical isolate used in this study is resistant to antifungal treatments commonly used in humans, the results of this study indicate that the antimicrobial photodynamic protocol ALAD-aPDT represents a valuable therapeutic approach that warrants further investigation for use against infections caused by *C. albicans.* ALAD-aPDT offers a potential solution to a significant drawback associated with conventional antifungal medication regimens. Antifungal medication treatments may become ineffective against fungal infections due to the development of antifungal drug resistance following exposure to antifungal agents. In contrast, the photodynamic mechanism produces ROS, which have a broad effect on several microbiological targets by causing the oxidation of microbial lipids, proteins, and carbohydrates. It could be interesting in subsequent research to investigate the exact mechanism that is triggered in *C. albicans.*

### 2.4. Membrane Fluidity

**Figure 4:**
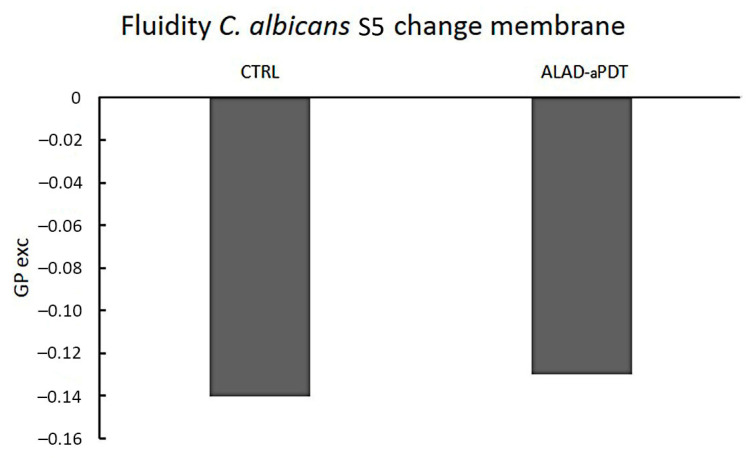
*Candida albicans S5* membrane fluidity changes after exposure to ALAD-aPDT using Laurdan generalized polarization (GPexc) values. The excitation GPexc was calculated using the following equation: GPexc = (I440 − I490)/(I440 + I490), where I440 and I490 are fluorescence intensities at 440 and 490 nm, respectively. Higher Laurdan GPexc values with respect to the control correspond to lower membrane fluidity. CTRL, control, unexposed *C. albicans S5*.

A possible role on the membrane fluidity modification induced by drug resistance has been recently suggested [24]. In this study, ALAD-aPDT did not affect *C. albicans S5* membrane fluidity with a GPexc value of −0.13 with respect to the control GPexc of −0.14 (*p* > 0.05) (Figure 4). The GPexc is a measure of membrane fluidity changes after the treatments indicating a modification of the degree of geometrical packing of the phospholipid membrane, a process governed by polar head group composition and fatty acyl chain conformation [25]. The lipid composition has been proven to influence drug delivery [26]. However, the photoinactivation of ALAD-aPDT may not be related to the organization of membrane phospholipids. This can be attributed to the intrinsic characteristics of ALAD gel, which is specifically formulated to make 5-ALA more easily overcome cell membranes. 5-ALA has a hydrophilic nature that impairs its passage through biological barriers. Thus, ALAD is a sol-gel formulated with a mixture of poloxamers to facilitate 5-ALA crossing lipidic membranes.

### 2.5. Cell Viability and Morphological Analysis of Gingival Fibroblasts

**Figure 5:**
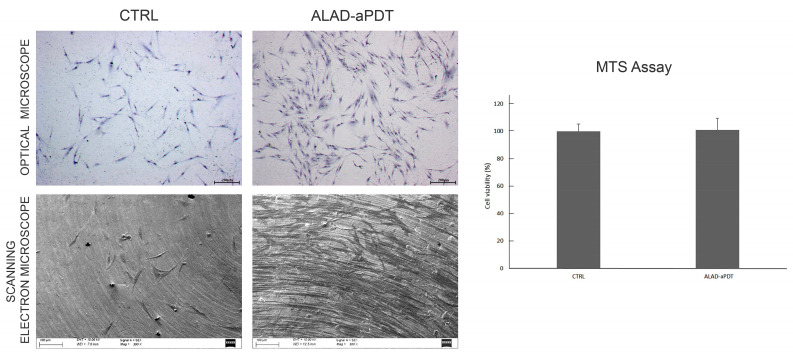
Morphological and cell viability analysis of hGFs treated with ALAD-aPDT. Observations using optical microscope (upper) and SEM (lower) were performed at magnifications of 25× (scale bar: 200 µm) and 300× (scale bar: 100 µm), respectively. Cell viability (right), evaluated at 24 h, was expressed in terms of percentage compared to CTRL.

The cell viability was evaluated using the MTS (2,5-diphenyl-2H-tetrazolium bromide) assay, which revealed no cell growth when hGFs were exposed to the ALAD-aPDT compared with non-treated cells (CTRL) (Figure 5). This demonstrated the absence of cytotoxicity. Previous research reported the proliferative abilities of fibroblasts subjected to photodynamic therapy [10,16,27,28]. In detail, Pierfelice TV et al. in 2022 showed that ALAD-aPDT significantly enhanced the proliferation rate at 48 and 72 h in gingival fibroblasts and oral osteoblasts [16]. In addition, Moore et al. observed that 5-aminolaevulinic acid did not negatively affect the viability of dermal fibroblasts irradiated with red light [27].

In addition, no morphological differences were observed in our study using optical microscopy and SEM (Figure 5). In both types of observations, the hGFs appeared spindle-shaped, and numerous interconnections characterized by philopodia were observed among the cells. However, the cell density increased in hGFs exposed to ALAD-aPDT compared with the CTRL. The preserved morphology of normal gingival fibroblasts after ALAD-aPDT treatment demonstrated the absence of cytotoxicity in this protocol. Jang YH et al. (2013) also studied the morphology of fibroblasts subjected to photodynamic therapy and observed no morphological changes [29].

**Figure 6:**
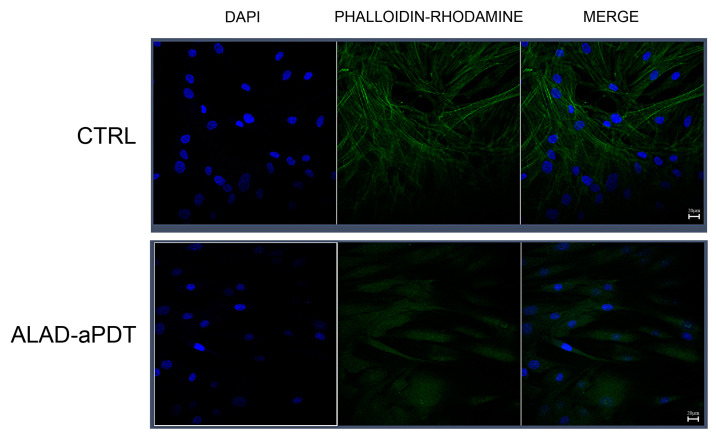
CSLM observations of hGFs exposed to ALAD-aPDT protocol at 24 h. DAPI staining (blue fluorescence) highlights the nuclei of cells; phalloidin/rhodamine staining (green fluorescence) shows cytoskeletal components. Magnification: 63×; scale bar: 20 µm.

To further study the morphology of cells, confocal laser scanner microscope (CLSM) evaluation was performed using DAPI and phalloidin (Figure 6). These stains were used to analyze the influence of ALAD-aPDT on the nuclei and filaments of actin. CLSM observations showed thickening actin filaments after ALAD-aPDT, as observed after phalloidin staining and after the merge of DAPI and phalloidin (Figure 6). This effect improved from the ALAD gel since it was metabolized in a photosensitizer, protoporphyrin IX [30]. In addition, the production of actin filaments continued after the generation of ROS; thus, the fluorescence intensity was higher in treated cells than in control cells [31].

### 2.6. ROS Levels Evaluation

**Figure 7:**
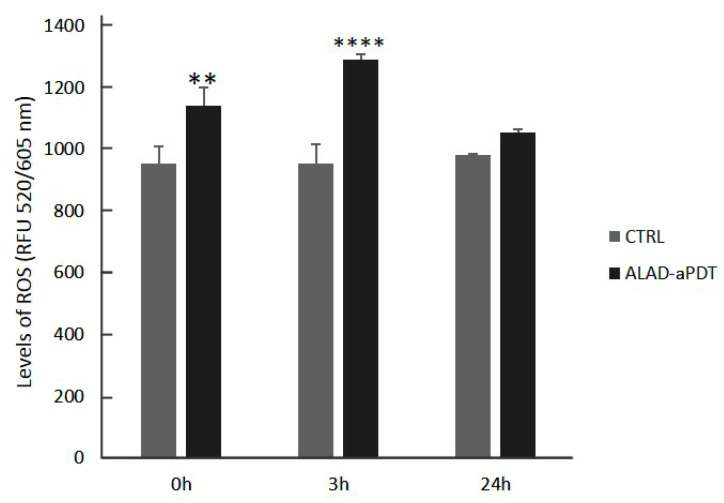
Levels of ROS evaluated in hGFs after the application of ALAD-aPDT protocol at 0 h (just after the end of the treatment), 3 h, and 24 h. (** *p* < 0.001; **** *p* < 0.0001).

The intracellular production of ROS was examined to confirm that the cultured fibroblasts internalized ALAD and generated intracellular ROS. The evaluation of reactive oxygen species (ROS) levels showed a significant increase in ALAD-aPDT just after the end of the treatment (0 h) (*p* < 0.001) and after 3 h (*p* < 0.0001) compared with the CTRL (Figure 7). However, the levels of ROS returned were comparable to the CTRL 24 h after the treatment. Wang P. et al. demonstrated that ALAD-aPDT stimulated the production of intracellular ROS as observed under fluorescence microscopy [32]. They also hypothesized that ALAD-aPDT occurred in the cytoplasm [32].

Currently, the mechanism of action of ROS is not clear. Some studies have observed that high levels of ROS can cause cell death, such as in cancer cells, but at same time, ROS were involved in intracellular signaling [33,34].

### 2.7. Evaluation of Collagen Production

**Figure 8:**
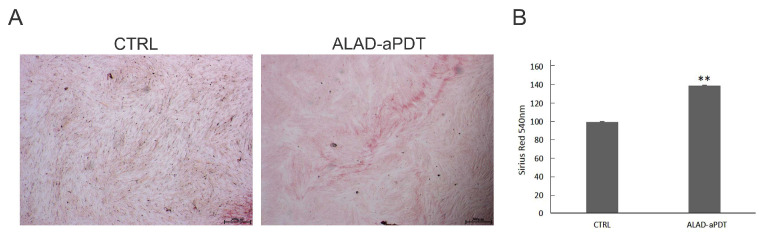
Picro-Sirius red staining observations (**A**) were quantified using spectrophotometric analysis at 540 nm (**B**). ** *p* < 0.001; Magnification: 25×; scale bar: 300 μm.

The collagen production was evaluated by Picro-Sirius red staining (Figure 8). The microscopy analysis, performed at 7 days, showed the presence of more intense red staining after the treatment with ALAD-aPDT compared to the CTRL (Figure 8A). The spectrophotometric analysis confirmed the qualitative results. The histogram showed a significantly higher production of collagen (*p* < 0.001) in the ALAD-aPDT group compared to the CTRL (Figure 8B). ALAD-aPDT stimulated collagen production just after 7 days, which was also demonstrated in in vitro and in vivo studies [32,35,36]. In detail, Wang P. et al. showed a clear increase in in type 1 collagen expression in fibroblasts [32]. In addition, photodynamic therapy was able to induce the upregulation of collagen production, as demonstrated by the increases in procollagen I mRNA, procollagen III mRNA, and procollagen I protein in patients [36].

## 3. Conclusions

In conclusion, this study showed the ability of ALAD-aPDT to significantly reduce *C. albicans* biofilm without any cytotoxic effects on gingival cells. Surprisingly, ALAD-aPDT induced an increased production of collagen by treated hGFs with respect to the controls.

## 4. Materials and Methods

### 4.1. Study Design

This study was conducted on an anonymized clinical resistant *Candida albicans S5* strain and human gingival fibroblasts (hGFs) to evaluate the antimicrobial potential on *C. albicans* and the absence of cytotoxicity on hGFs under a photodynamic protocol based on using a gel containing 5% of 5-aminolaevulinic acid (ALAD; ALPHA Strumenti s.r.l., Melzo, Milan, Italy) and a red LED light (PDT; 630 nm) (ALPHA Strumenti s.r.l.). The LED device showed a tip diameter of 6 mm, an irradiance surface of 380 mW/cm^2^, and a specific dose of 23 J/cm^2^ for each minute. The protocol provided ALAD for 45 min, followed by the irradiation with LED for 7 min. During the experiments, the LED handpiece was mounted perpendicularly to the wells at a distance of 0.5 mm, with a particular polystyrene box to maintain a constant distance from the light source.

For this study, the untreated cells were considered as the control (CTRL). Cells irradiated without ALAD and cells incubated with ALAD in the dark were not inserted into this study, because these conditions have already been tested on bacteria, and on normal and pre-cancerous cells in previous research [11,16,37,38,39]. In these studies, the viability of cells treated with ALAD gel without irradiation was perfectly comparable with the viability of untreated cells. These results suggest that ALAD gel requires photoactivation to exert its effect. In previous studies, LED irradiation did not show any effects on human normal cells, and a slight capability (~10%) to reduce the viability of different microorganisms and eukaryotic cells [10,11,15,16,37].

The effects of ALAD-aPDT on *C. albicans S5* biofilm were evaluated in terms of the following:

− biofilm biomass evaluation by Hucker’s crystal violet staining method;
− the colony forming units (CFU/mL) count for the quantification of cultivable cells;
− cell viability via live/dead analysis;
− membrane fluidity changes.

The microbiological experiments were conducted in triplicate.

The effects of ALAD-aPDT on hGFs were performed as follows:

− Cell viability at 24 h;
− Morphological analysis via SEM and optical microscopy at 24 h;
− Laser scanning microscope analysis at 24 h;
− ROS levels at 0, 3, and 24 h;
− Collagen production using Picro-Sirius red staining.

### 4.2. Microbiological Analysis

#### 4.2.1. Fungal Culture and Biofilm Preparation

A clinical strain of *Candida albicans S5*, isolated from the oral cavity of healthy individuals and collected at the Department of Medical, Oral, and Biotechnological Sciences, was used for this study (reference number: BONEISTO N. 22-10.07.2021, University G. d’Annunzio Chieti-Pescara, 10 July 2021, approved by the Inter Institutional Ethic Committee of University “G. d’Annunzio” Chieti-Pescara, Chieti, Italy). The resistance profile of *C. albicans S5* towards antifungal drugs commonly used in therapy was analyzed. The strain for the experiments was grown on Sabouraud dextrose agar (SAB, Oxoid, Milan, Italy), cultured in RPMI 1640 (Sigma-Aldrich, Milan, Italy) plus 2% glucose, and standardized to obtain a suspension containing ≈10^7^ CFU/mL, optical density/OD_600_  =  0.15, as performed using a spectrophotometer (Eppendorf, Milan, Italy).

For the biofilm preparation, 200 µL of standardized *C. albicans S5* suspension was incubated on 96-well flat-bottomed microtiter plates at 37 °C for 24 h [12]. Subsequently, the wells were rinsed three times with sterile PBS to remove the planktonic cells, and then treated with ALAD-aPDT as described.

#### 4.2.2. Biofilm Biomass Assay

After treatment with ALAD-aPDT, the biofilm biomass (comprising bacterial cells/extracellular polymeric substances) was quantified via staining dry biofilms with crystal violet 0.1% (Sigma Aldrich, Milan, Italy) for 1 min. Then, the biofilms were washed with PBS to remove excess stain and dried for 2 h at 37 °C. The crystal violet was eluted with ethanol, and biofilm formation was then measured via absorbance assessment at 570 nm with a microplate reader (SAFAS, Munich, Germany).

#### 4.2.3. Colony Forming Units Count

The CFU/mL determination was performed as follows: after the treatment, the adhered *C. albicans S5* cells were scraped off, resuspended in 200 μL of PBS into test tubes, and vortexed for 2 min. Serial dilutions of 1:10 were spread on SAB, and then incubated for 24–48 h at 37 °C.

The number of CFU/mL was determined by calculating the average count of three agar plates for dilution.

#### 4.2.4. Viability Test

Microscopic observations using live/dead staining BacLight viability kits were performed to evaluate the viability of *C. albicans S5* cells in the biofilm, with and without (control) ALAD-aPDT treatment. A total of 10 fields of view each, in a random way, were examined by three blinded microbiologists for the green (viable cells) and red (cells with impaired membrane activity) cellular fungal amount.

#### 4.2.5. Membrane Fluidity

Laurdan-generalized polarization (GPexc) was used to detect the ALAD-aPDT treatment effect on *C. albicans S5* membrane fluidity [25]. Briefly, the standardized broth culture was exposed to the ALAD-aPDT, and then washed twice with 15 mM Tris–HCl buffer (pH 7.4), resuspended with 10 µM Laurdan, incubated for 1.5 h in the dark at 37 °C, and then analyzed. The samples were analyzed using a Varian Cary Eclipse fluorescence spectrofluorometer (Agilent Technologies, Santa Clara, CA, USA) to evaluate the Laurdan emission. The excitation GPexc was calculated using the following equation: GPexc = (I440 − I490)/(I440 + I490), where I440 and I490 are fluorescence intensities at 440 and 490 nm, respectively. Higher Laurdan GPexc values with respect to the control correspond to lower membrane fluidity.

### 4.3. Cellular Analysis on Mammalian Cells

#### 4.3.1. Cell Culture

hGFs were purchased from ATCC (Manassas, VA, USA) and cultured using DMEM low glucose (Corning, New York, NY, USA) supplemented with 10% fetal bovine serum (FBS) (SIAL, Rome, Italy), 1% penicillin, and streptomycin (Corning) at 37 °C and 5% CO_2_.

#### 4.3.2. Cell Viability

The cell viability was evaluated at 24 h using the CellTiter96 assay (3-(4,5-dimethylthiazolyl-2)-2,5-diphenyltetrazolium bromide; MTS, Promega, Madison, WI, USA). Cultures of 1 × 10^4^ cells/well were grown in 96-well plates, and were exposed to the ALAD-aPDT protocol. A volume of 10 µL of MTS solution was added to each well. A microplate reader (Synergy H1 Hybrid BioTek Instruments, Hampton, New Hampshire, USA) was used to read the optical density at 490 nm. The cell viability was evaluated as a percentage compared to non-treated cells (CTRL).

#### 4.3.3. Optical Microscope Analysis

In 24-well plates, 2∙10^4^ cells/well were seeded and treated with the ALAD-aPDT protocol. After 24 h, the cells were fixed using 70% cold ethanol and stained with 1% toluidine blue and 1% borax (Sigma Aldrich). The cells were observed using a stereomicroscope connected with a camera at 25× (Leica, Wild Heer-brugg, Wetzlar, Germany).

#### 4.3.4. Scanning Electron Microscope

The morphology of hGFs was also evaluated using a scanning electron microscope (SEM). Titanium dental implant discs (Implacil, DeBortoli, São Paulo, Brazil) were used as a surface to culture the cells, where 2∙10^4^ cells/well were seeded on the discs and were exposed to ALAD-aPDT. After 24 h, the samples were fixed with 2.5% glutaraldehyde for 1 h, dehydrated using sequential concentrations of ethanol, and sputtered with gold. They were observed under 300× magnification using an SEM (EVO 50 XVP LaB6, Carl Zeiss, Cambridge, UK) at 10 kV.

#### 4.3.5. Confocal Laser Scanning Microscope (CLSM)

hGFs were cultured in 8-well culture glass slides (Corning, Glendale, AZ, USA) at a density of 1.3 × 10^4^/well, and treated with ALAD-aPDT. After fixation with 4% of paraformaldehyde (PFA) (BioOptica, Milan, Italy) in 0.1 M PBS (Lonza, Basel, Switzerland), the cells washed three times in PBS and permeabilized with 0.1% Triton X-100 (BioOptica) in PBS for 5–6 min. The cytoskeletal actin and the nuclei were stained, respectively, with rhodamine-phalloidin (Invitrogen, Waltham, MA, USA) and DAPI (4′,6-diamidino-2-phenylindole dihydrochloride; Sigma, St Louis, MO, USA), both prepared at 1:1000 in PBS and maintained for 1 h at 37 °C. The images were acquired through the Zeiss LSM800 confocal system (Carl Zeiss, Jena, Germany).

#### 4.3.6. ROS Levels

In 96-well plates, 10^4^ cells/well were seeded to measure the ROS levels. After 24 h, the hGFs were exposed to the ALAD-aPDT protocol. According to the manufacturer’s protocol, ROS levels were measured at 3, 6, and 24 h after the treatment using the cellular reactive oxygen species detection assay kit (Abcam, Cat No. ab186027, Cambridge, UK). The fluorescence at λ ex/em 520/605 nm was measured with a microplate spectrofluorometer (Synergy H1 Hybrid BioTek Instruments).

#### 4.3.7. Picro-Sirius Red Staining and Spectrophotometric Analysis

hGFs were cultured in 24-well plates at a density of 5∙10^4^ cells/well, and treated with ALAD-aPDT; after 7 days, the cells were fixed with glutaraldehyde 2.5% for 2 h. Then, the cells were incubated with the staining solution (Sigma Aldrich) at room temperature for 1 h, and the hGFs underwent three rounds of acetic acid washing (0.1% concentration). The images were captured using a stereomicroscope (Leica) at 25× magnification. Picro-Sirius red was eluted in 0.1 N sodium hydroxide for 1 h, and then a spectrophotometric examination was performed, reading the optical density (OD) at 540 nm with a microplate reader (Synergy H1 Hybrid BioTek Instruments).

### 4.4. Statistical Analysis

Statistical analyses were performed with GraphPad 8.0.2.263 (GraphPad, San Diego, CA, USA) software, utilizing one-way ANOVA followed by post hoc Tukey’s multiple comparisons analysis. Values of *p* < 0.05 were considered statistically significant.

## Data Availability

The data presented in this study are openly available in the article.
